# Supplementary material for: The influence of background music and narrative setting on anthropomorphic judgements of killer whale (Orcinus orca) emotional states and subsequent donation behavior
Source: PLoS One. 2023 May 24;18(5):e0282075. doi: 10.1371/journal.pone.0282075 (PMC10208518; doi:10.1371/journal.pone.0282075)
Supplement: S1 Text — (DOCX) [file pone.0282075.s016.docx]

**Supplemental Material**

**Pilot Study**

***Pilot Study***

A pilot study was conducted with a convenience sample using a snowball technique to test the video clip, music manipulation, randomization, and questions. The details of the pilot study are presented in this supplemental material section. In brief, the pilot study, which recruited university students and their peers (*n* = 116) from three different private, liberal arts colleges and universities, indicated that the video clip viewed by the participants influenced responses and needed to be replaced with something more neutral. Moreover, in the pilot study four types of music (i.e., happy, sad, angry, aquatic noises) were contrasted to a silent control. All music excerpts, except the aquatic noise, had previously been validated as effective emotionally inducing stimuli [32]. Participants were more likely to perceive the killer whale as experiencing no emotion or neutral emotion when hearing an aquatic noise (i.e., neutral sound control) as opposed to no background sound (no sound control). Happy and sad music produced expected emotional attribution outcomes, but angry music elicited a broad range of emotional attributions. Given that the free responses provided in the control aquatic noise condition were more ecologically valid and most documentaries do not have silent background soundtracks, we presented the aquatic sound as our control rather than complete silence in the subsequent experiments because ecological validity was enhanced. Additionally, the angry music condition was dropped for the last two experiments after testing it with the new video clip in Experiment 1 and replicating the ambiguous results found in the pilot study.

As described in the materials and measures section, a pilot study was conducted to evaluate the effects of music on the emotional appraisal of a single killer whale. The method and results are described below.

***Materials and Measures***

*Participants*

Using a snowball-based technique for recruitment at three private, small primarily undergraduate institutions, 116 participants (75% females), between the ages of 18-60+ (84% 18-22 years), completed the study surveys. The participants primarily resided in the following regions: North, West, Midwest, Northeast, Southwest, and Southeast. Course credit was received, if needed, for participating in survey. Most had not seen *Blackfish* (53%). IRB approval was granted by St. Mary’s University and all participants had the opportunity to provide written informed consent, leave the study at any time, and were debriefed at the end of the survey.

*Materials and Measures*

A video clip of a rocking, but stationary, orca presented was used to create five different videos that differed by types of background music (happy, sad, angry, silence, and aquatic noises. Video editing was performed in Filmora Wondershare. These videos were embedded into a Qualtrics-based survey, which included questions regarding their demographics, beliefs about animals, and hypothetical donation behavior.

*Procedure*

Participants were recruited from other undergraduate universities through colleagues of HH. Two research assistants created five 20-sec video clips and selected music from the findings of Lepping et al. (2016). The video clips were embedded within a Qualtrics-based survey that were randomly disbursed to each participant following a block randomization procedure. The survey took participants 3-6 minutes to complete, depending on responses as both free response and forced choice options were provided for each measure of interest.

***Results***

A chi-square test of independence indicated a significant relationship between background music and emotion of the killer whale, χ²(16, *N* = 116) = 33.05, *p* = .007, Cramer’s V = .27. Significantly more participants identified the whale as happy when they heard happy music (42%, *n* = 18), identified the whale as sad when they heard sad music (36%, *n* = 10), identified the whale as neutral when heard aquatic noises (8%, *n* = 3), and identified the whale as neutral when heard no background music (41%, *n* = 15). Angry music produced a number of different emotional interpretations.

A 2-way mixed ANOVA indicated that donation percentage differed by charity, *F*(1.97,187.18) = 6.88, *p* = .001, ɳ² = .07, but music did not appear to influence the donation behavior despite the higher percentages for happy and sad music. Participants indicated that they would spend a significantly higher amount of their earnings for Supporting Wild Orcas (40% ± 29%) as compared to Supporting Captive Orcas (22% ± 22%).

***Discussion***

Results from both the pilot and the national samples found significant effects of music on the interpretation of the killer whale’s current emotional state. Happy music tended to produce happy whales; sad music produced sad whales; neutral music produced neutral whales. These results extend those of a previous study performed with sharks [14]. Participants reported that the whale was happy when hearing the angry music, which may have been related to the music selection and the video clip used. This information was utilized in the first study such that a different music selection was utilized for the angry clip. Finally, hypothetical donation behavior was measured in the pilot study and resulted in participants being more likely to donate a higher percentage of their earnings to wild orca research as compared to captive orca research. The hypothetical donation behavior was not significantly influenced by the background music experienced, although a larger sample might produce significant results. Although almost half of the pilot sample had viewed “Blackfish” prior to the study, this experience was not significantly related to their emotional rating or to their hypothetical donation behavior.
