# Supplementary material for: The influence of background music and narrative setting on anthropomorphic judgements of killer whale (Orcinus orca) emotional states and subsequent donation behavior
Source: PLoS One. 2023 May 24;18(5):e0282075. doi: 10.1371/journal.pone.0282075 (PMC10208518; doi:10.1371/journal.pone.0282075)
Supplement: Attachment — Submitted filename: BackgroundmusicPlosOne_REVIEWERCOMMENTS-01172023.docx [file pone.0282075.s017.docx]

Background Music Reviewer Comments

Reviewer #1: I think the topic of this paper is quite interesting and the front-end of paper (up to general materials and methods) was quite well written and relatively easy to follow. But the authors still failed to provide clear hypotheses and directions of their prediction, which made the whole story to be a bit ambiguous and lack of big theoretical contribution.

Moreover, I have some major concerns on the materials and methods sections.
1. p. 10, it was not quite clear what were the "several quality control checks" (line 212).
2. p. 11, the authors used a set of critiera to exclude a high percentage of date (up to 22% and 41%), which is not acceptable in most experimental research. I would suggest the authors to find ways to decrease the number of the problematic data and re-collect all the data in all the studies. If all experiements have such a high percentage of bad data, the experiment per se could be problematic and the authors should be responsible to find out the reasons and solve these problems, instead of excluding all the bad data simply.

The data was collected using MTurk, and as Chmielewski & Kucker (2020) note the declining response quality from the Summer of 2018 onwards meant that even well-established findings could not be replicated. This is not however a reflection of the quality of the experimental design, but of the emergence of bots, virtual private servers (VPS) and other means that have allowed fraudulent access to MTurk studies. In fact, Chmielewski & Kucker suggest taking extra care to ensure the validity of responses. An analysis of 2018 responses found that 20% of responses were coming from a VPS or a non-US IP address, which reflects individuals fraudulently gaining access to surveys meant for US participants in order to capitalize on survey payment (Kennedy et al., 2020). Our experiment follows best practices in online data collection (brevity and simplicity), and we do not think that re-collecting the data or changing the experimental design would improve the validity of the findings. In fact, until technology improves, the only method we have to ensure the validity of the data is to exclude as much bad data as we can identify.

We have included a diagram that shows the controls implemented in each experiment and where participants were lost (e.g. sound off etc).

Chmielewski, M., & Kucker, S. C. (2020). An MTurk Crisis? Shifts in Data Quality and the Impact on Study Results. *Social Psychological and Personality Science*, *11*(4), 464–473. <https://doi.org/10.1177/1948550619875149>

Kennedy, R., Clifford, S., Burleigh, T., Waggoner, P. D., Jewell, R., & Winter, N. J. G. (2020). The shape of and solutions to the MTurk quality crisis. *Political Science Research and Methods*, *8*(4), 614–629. <https://doi.org/10.1017/psrm.2020.6>

3. It is unclear why the authors choose four types of music (p. 11), especially the aquatic noises.

We have edited the text to clarify why the four types of music were selected, but the reasoning included information from a pilot study that was conducted originally based on literature and the study that was being extended (Nosal et al. 2016).

4. Even after excluding a large percentage of data, the statistical results were still not highly significant, and most results were still not significant (e.g., p. 15, p=.054, and p=.098), which are not consistent with what the authors proposed in the frond-end. There are quite a number of different places that have mixed results that were not consistent with predictions at the front end or have no predictions or theories to expect the results (p. 21).

Given that this study had only one other study from which to base hypotheses from, many of the predictions were extensions from multiple sources of information. Nosal et al also found a small effect with a sample size that was even larger ultimately than our sample size. Our findings indicated that people were influenced by music in *two* of the conditions. Monetary extension was a small finding. We have edited our text for clarity to clarify the concerns about mixed results.

5. The authors also found that there is no interaction effect between music and setting (p. 24), which made the whole story at the beginning to fall apart further.

While the interaction did not occur as we anticipated (which again was a prediction made from multiple sources, other important findings did emerge and supported our original predictions. We also discovered that the findings were mediated by beliefs held about the welfare of the animals, thus the picture was more complex than we had originally predicted. We have attempted to clarify these points in our revision.

6. The authors found no direct effects on emotion appraisals on donation behavior (p. 25), and the results were also not consistent with their prediction (p.27), which basically contradicted what they have said before.

We have edited our results presentation to clarify the confusion associated with this comment. Donation behavior did change based on a complex mediation of welfare beliefs and music for wild animals. We are comfortable with our science and the findings that did not support the original predictions as they were based on one study and a combination of multiple sources that have not looked at the topic we chose to examine.

Overall, because the experimental methods and the data have some serious problems, the results are not consistent and reliable enough to draw any supportive conclusions. I would suggest the authors to find out a strong theoretical support for their predictions, and re-run all the experiments using a much better way of collecting data to avoid excluding a high percentage of data.
Good luck for the authors to continue on this interesting topic.

Thank you for your thorough review of our experiments. We hope that our revisions have clarified some of the concerns outlined above.

Reviewer #2: General comments:

- This is an interesting paper that is generally well written; however it needs a bit of polishing and clarification in some parts (see specific comments, which are minor, below).
- Overall the paper seems to run a bit long (i.e., the writing could be punchier) and could probably be shortened by about 10-20%.
- Some parts of the paper seem repetitive, which could probably be lessened with some restructuring/reorganizing.

We have attempted to edit our text heavily to reduce redundancy and reorganize our thoughts.

Specific Comments:

19: Consider identifying the gap in knowledge (that your study fills before diving into the study itself).

We identified gaps in knowledge in our revision.

20: Is emotion referring to human subjects or whales? Unclear as written. Based on title and later sentences, I believe it is the whales’ emotional state.

We have gone through our text and specifically identified the terms we wished to represent the nature of the study. It is centered around the whale’s emotional state, not the human. That is the unique contribution of our study.

42-43: Be more specific (or provide some examples) of significant legislative change

We have removed this information as it distracted from the goal of the paper.

45: Over what period did the stock plunge? Should be more specific because even with the pandemic, it is currently trading at all-time highs.

We removed this information from the revision as it distracted from the goal of the paper.

47-53: Like the comment in Abstract, I think the intro would benefit from identifying the gap in knowledge that this study fills. What was not known that this study addresses? In other words, it’s not clear what the impetus for the study is.

We have edited the intro to address the gap in knowledge.

161: Could you be more specific about how you expected the two factors to interact?

We have edited the text to address our expectations and added several figures to illustrate the ideas within the method and results.

214: Choose better word than neutral…that makes me think the background was somehow edited out and replaced with a uniform gray (or similar) background.

We have revised the language to clarify our intent of this condition.

222: Not sure what ‘neutral’ means here. Maybe ‘ambiguous’ is better?

We have revised the language as suggested.

330: Was it the same exact footage, only differing only by background music? This sounds like it was 4 different footage, which were in turn each randomly paired with one of the 4 background music selections.

We have clarified this information with edits and additional figures.

415: “orca in the marine water oceanarium” is different from what was mentioned above. Also, why “the” oceanarium as opposed to “a” or “an”?

The statement is what we used in the clip viewed by participants. “the” was used to match the format in phrasing to control for any influence by word length or word choice.

421: When were participants asked to recall the setting? Was this before or after the emotion questions? If before, I would be concerned that drawing attention to the setting might clue respondents into what the researchers were testing.

We have added several figures to clarify the order of questions for each experiment, but it was at the end of the survey after the critical questions.

435: Did you include all respondents, even ones who did not remember the supposed setting? Or did you only include respondents that correctly remembered the setting?

For the experiments in which setting was a critical manipulated factor, the question acted as an attention check, and if it was missed, the participants were not included in that analysis. For the experiment in which setting was not manipulated, all individuals were included, regardless of what they though the setting was.

517: Should use the same descriptive words for the different settings. What is “aquatic” in this case? Previously I believe you called this “neutral”. Also, here it is clear what 3x3 means, but earlier 3x3 confused me because you mentioned 4 music conditions (including angry). Here it is clear you omitted “angry” but that was not clear earlier.

We have revised the text to clarify these questions. Angry was used in the initial pilot study (supplemental material) and in the first study to ensure that our findings from the pilot study were consistent after we modified our video selection of the killer whale. Angry music is reported for Experiment 1 but not 2 and 3. This is also visible in our figures outlining the experimental protocol for each study.

519-520: I don’t quite understand the “rule” in this sentence. Please clarify.
525: Here the “neutral” setting condition is called “aquatic/control”. This is just a general comment throughout, to keep the terminology of the different experimental conditions of the videos consistent and define them clearly at first use.
569: Not sure what “reverse coding is”. Maybe it was defined above, but I don’t remember.

We have attempted to clarify all the questions raised in these three points. We codified our language regarding the control condition across each study and defined rule and reverse coding.

801: Did you ask participants whether they had seen Blackfish? If not, how are you making this assertion that most had not seen it? Regardless, I agree that statement is probably true.

We did ask the participants whether they had viewed Blackfish, The Cove, and other films.

Table 1: Comma after the word “calm” suggests there was more to this open-ended response? If not, delete comma. In general I think the examples of responses should be put in quotes.

We have added quotes. All punctuation was provided by the participant referenced.

Fig 1, Experiment 3: $0.50 “if completed study” reads a bit strange…how is this different from the other two experiments? I understand the $0.50 was basically to give the respondents an opportunity to donate that amount, so I think that should be included here for clarity.

We have edited our figures to clarify this question.

Fig 2: The stippling pattern for happy bars is a bit hard to read. Consider just using three different shades of solid gray/black for the three conditions, like Fig 3. Also, the “note” says the bars represent “number” of participants, but it is actually the proportion/frequency.

Adjusted the fill pattern for the three conditions and clarified the note to reflect frequency of participants (not proportion).

Fig 3: Generally fine, but the error bars look nearly identical for all bars. Just double-check these were added correctly to graphs and that the actual SEs are very similar, as suggested by the error bars. Also, the patterns are a bit hard to distinguish. As above, consider using 4 different shades of solid gray/black instead.

Standard errors indeed range only from .31 to .36, so they are actually very similar across the 9 experimental conditions. Figure has been adjusted so that the bars are solid shades of gray and black that reflect the three levels of the Video Setting factor.

Figs 1-3: Consider displaying sample sizes N for the different conditions.

We have modified the caption to reflect the sample sizes for relevant figures.
